# Design of Thymidine Analogues Targeting Thymidilate Kinase of *Mycobacterium tuberculosis*

**DOI:** 10.1155/2013/670836

**Published:** 2013-03-24

**Authors:** Luc Calvin Owono Owono, Melalie Keita, Eugene Megnassan, Vladimir Frecer, Stanislav Miertus

**Affiliations:** ^1^Laboratory for Simulations and Biomolecular Physics, Advanced Teachers Training College, University of Yaoundé I, P.O. Box 47, Yaoundé, Cameroon; ^2^Centre for Atomic Molecular Physics and Quantum Optics (CEPAMOQ), University of Douala, P.O. Box 8580, Douala, Cameroon; ^3^International Centre for Science and High Technology, UNIDO, Area Science Park, Padriciano 99, 34012 Trieste, Italy; ^4^Laboratoire de Physique Fondamentale et Appliquée, Université d'Abobo-Adjamé, 02 BP 801 Abidjan 02, Cote d'Ivoire; ^5^Cancer Research Institute, Slovak Academy of Sciences, 83391 Bratislava, Slovakia; ^6^Department of Physical Chemistry of Drugs, Faculty of Pharmacy, Comenius University, 83232 Bratislava, Slovakia; ^7^International Centre for Applied Research and Sustainable Technology (ICARST), 81404 Bratislava, Slovakia; ^8^Faculty of Natural Sciences, University of Ss. Cyril and Methodius, 91701 Trnava, Slovakia

## Abstract

We design here new nanomolar antituberculotics, inhibitors of *Mycobacterium tuberculosis* thymidine monophosphate kinase (TMPK*mt*), by means of structure-based molecular design. 3D models of TMPK*mt*-inhibitor complexes have been prepared from the crystal structure of TMPK*mt* cocrystallized with the natural substrate deoxythymidine monophosphate (dTMP) (1GSI) for a training set of 15 thymidine analogues (TMDs) with known activity to prepare a QSAR model of interaction establishing a correlation between the free energy of complexation and the biological activity. Subsequent validation of the predictability of the model has been performed with a 3D QSAR pharmacophore generation. The structural information derived from the model served to design new subnanomolar thymidine analogues. From molecular modeling investigations, the agreement between free energy of complexation (ΔΔ*G*_com_) and *K*_*i*_ values explains 94% of the TMPK*mt* inhibition (*pK*_*i*_ = −0.2924ΔΔ*G*_com_ + 3.234; *R* ^2^ = 0.94) by variation of the computed ΔΔ*G*_com_ and 92% for the pharmacophore (PH4) model (*pK*_*i*_ = 1.0206 × *pK*_*i*_^pred^ − 0.0832, *R* ^2^ = 0.92). The analysis of contributions from active site residues suggested substitution at the 5-position of pyrimidine ring and various groups at the 5′-position of the ribose. The best inhibitor reached a predicted *K*_*i*_ of 0.155 nM. The computational approach through the combined use of molecular modeling and PH4 pharmacophore is helpful in targeted drug design, providing valuable information for the synthesis and prediction of activity of novel antituberculotic agents.

## 1. Introduction

A substantial number of the influenza A subtype H1N1 death cases reported by WHO occurred in patients with chronic respiratory conditions shedding light on possible impact of influenza on active tuberculosis (TB) patients [1]. Tuberculosis kills more than 2 million people [2] and infects around 2 billion worldwide [3] with more than 9 million cases annually [4]. According to WHO in the second millennium decade (2020), over 1 billion people will be newly infected and 36 million will die from TB [5, 6] making it a leading cause of mortality as infectious disease. In this regard, the millennium development goal (MDG) to halve by 2015 TB mortality relative to the 1990 level is problematic [7]. First, mortality in comparison with the 1990 level is slightly decreasing but not in Africa [2]. Secondly, the increased occurrence of MDR and XDR-TB strains is disqualifying the current 40-year-old and long-term DOTS drugs: isoniazid, rifampicin, pyrazinamide, and ethambutol [2]. Accordingly the need of new low-cost and short-term anti-TB therapies is more than urgent regardless those currently in the preclinical or early clinical phase since most of them are improvements on existing antimicrobial compounds with nonnegligible susceptibility to resistance.

The development of new drugs is entirely subordinated to the fulfillment of a particular profile: potency and rapid action short-term DOTS efficiency against MDR-TB safer than existing treatment coadministrable with anti-retrovirals easy to use in the field-action against latent as well as active forms [8]. Another requirement is the increased efficacy on targets relevant to dormancy phase and preventing from “nonproliferation and back resuscitation into growth phase” [3].

In the forthcoming of the availability of appropriated new targets, attention is given to enzymes catalyzing vital processes such as NAD supply [9] or ATP-dependent phosphorylation [10]. TMPK*mt* is the last specific enzyme for the synthesis of dTTP catalyzing dTMP conversion into dTDP using ATP as phosphoryl donor making its inhibitors potential anti-TB drugs targeting DNA replication [11]. So far the most potent thymidine-like inhibitors reported are in low micromolar range (3.5–5 *μ*M) activity. The lack of leads for TMPK*mt* inhibitors and the availability of X-ray crystals structure of the enzyme bound to substrate open the gate to the design of new antitubercular agents with this scaffold [12].

**Figure 1:**
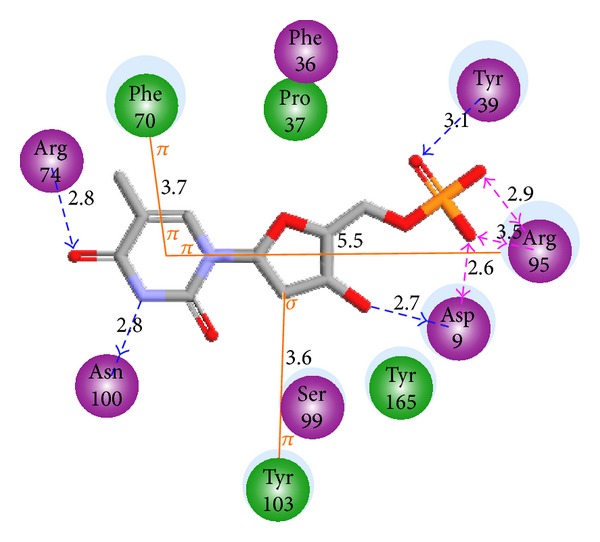
Interaction of dTMP with active site residues of TMPK*mt*.

The high-resolution 3D structures of TMPK*mt*-dTMP complexes (*K*_*i*_ = 4.5 *μ*M) have been released at 1.6 Å-1GSI [13] and 1.9 Å-1G3U [14]. The first one with the highest resolution is displayed as 2D interaction view in Figure 1 depicting the interactions of thymine pyrimidine ring: stacking with Phe70 (*d* = 3.7 Å) and cation-*π* with Arg95 (*d* = 5.6 Å). Hydrogen bonds with Arg74 and Asn100 keep the orientation of the pyrimidine ring while the ribose ring's hydroxyl is bonded to Asp9. The orientation of Arg95 is also kept by its HB to one of the phosphate oxygen atoms; this polar group interacts with Tyr39 and the Mg^2+^ ion. 

On the basis of these interactions, dTMP analogues have been reported by removing the monophosphate counterpart and Br substitution on the pyrimidine ring resulted in keeping the potency of the substrate [15] opening an important gate since the polarity of the dTMP analogues degrades their ADME profile due to their inability to cross cell-membrane [16]. Focusing on nucleosides instead of nucleotides with a hydroxyl group in place of OPO_3_^2−^ for dTMP resulted in a sixfold drop of activity but a simple bromine substitution in the 5-position of the pyrimidine ring kept the initial potency (TMD4, *K*_*i*_ = 5 *μ*M) [15]. This position is not suitable for other halogens F-212 *μ*M, Cl-10 *μ*M, and I-33 *μ*M neither for hydroxyls OH-270 *μ*M, CH_2_OH-820 *μ*M nor alkyl chains CH_3_CH_2_-1140 *μ*M as repulsion was expected in the case of groups larger than CH_3_  [15]. No phenyl ring has been tried in that position for this nucleoside but a sevenfold decrease in potency is observed in the case of dTMP with a benzyl (C_6_H_5_CH_2_-28 *μ*M) [16]. The stabilizing feature at the 5-position of the pyrimidine ring is not purely hydrophobic, and an orientational part has to be considered. For this we explore here the possibility to design analogues with different small groups in place of the above cited groups bearing both requirements.

The 2′ position on the ribose ring has been occupied by hydroxyl and halogens with no specific improvement of potency neither. Various substitutions in 3′ did not improve the potency. Differently the 5′-position is the second enriching one after that on the pyrimidine ring. Few substitutions have been made beside OH : N_3_, NHCOCH_3_, NH_2_, and halogens usually to keep the interactions involving the phosphate group in dTMP analogues. More recently attempts to replace the ribose ring with a phenyl [17] with no real improvement followed by a bicyclic sugar derivatives [18] reaching 3.5 *μ*M and later by a spacer ended by acyclic nucleoside analogues exploring edge to face interaction between the naphthyl group and Tyr39 has led to 0.27 *μ*M potency [19]. A Computer-assisted combinatorial design of bicyclic thymidine analogs as inhibitors of TMPK*mt* by Frecer et al. [20] identified submicromolar concentration range inhibitors with favorable ADME profile.

In the study reported herein the thymidine scaffold is kept with substituted small size group at position 5 of the phenyl ring, 2′, 3′ on the ribose ring and finally at 5′-position taking in to account the structure of the training set (TS). A QSAR model of interaction with TMPK*mt* was built from a TS of 15 TMDs starting from the above mentioned 1GSI.pdb 3D structure to compute the free energy of complexation taking into account the interaction energy, the solvation free energy, numeric solution of the Poisson-Boltzmann scheme and finally the conformational entropy variation of the inhibitor upon binding. The predictability of the model was further crossed with a PH4 3D QSAR one used to screen a library of thymidine analogues (TMAs) for subnanomolar range analogues. The identified hits from the complexation QSAR equation finally reach a predicted activity in the picomolar concentration range.

## 2. Material and Methods

### 2.1. Training and Validation Sets

The training and validation sets of thymidine analogues inhibitors of TMPK*mt* used in this study were selected from the literature [15–17]. The inhibitory potencies of these derivatives cover sufficiently broad range of activity to allow a reliable QSAR model to be built (5 ≤ *K*_*i*_^exp⁡^ ≤ 1900 *μ*M).

### 2.2. Model Building

Molecular models of the enzyme-inhibitor complexes (*E* : *I*), free TMPK*mt* (*E*) and inhibitors (*I*) were prepared from high-resolution crystal structure of the reference complex containing the deoxythymidine monophosphate TMPK*mt* : dTMP [13] (Protein Data Bank [21] entry code 1GSI at a resolution of 1.6 Å) using Insight-II molecular modeling program [22]. The structures of the *E* and *E* : *I* complexes were considered to be at a pH of 7 with neutral N- and C-terminal residues and all protonizable and ionizable residues charged. No crystallographic water molecule was included into the model. The inhibitors were built into the reference structure complex by *in situ* replacing of the derivatized R-group of the dTMP moiety (scaffold). An exhaustive conformational search over all rotatable bonds of the replacing function group, coupled with careful gradual energy minimization of the modified inhibitor and the TMPK*mt* active site residues located in the vicinity of the inhibitor (within 5 Å distance), was employed to identify the low-energy bound conformations of the modified inhibitor. The resulting low-energy structures of the *E* : *I* complexes were then carefully refined by minimization of the whole complex. This procedure has been successfully used for model building of viral and protozoal protease-inhibitor complexes and design of peptidomimetic and hydroxynaphthoic inhibitors [23–27].

### 2.3. Molecular Mechanics

Simulations of the models of inhibitors, TMPK*mt,* and their complexes were carried out with all-atom representation using atomic and charge parameters of the class II consistent force field CFF91 [28]. A dielectric constant of 4 was used for all molecular mechanics (MM) calculations in order to take into account the dielectric shielding effect in proteins. Minimizations of the *E* : *I* complexes, free *E* and *I* were carried out by relaxing the structures gradually, starting with added hydrogen atoms, continued with residue side chain heavy atoms and followed by the protein backbone relaxation. In all the geometry optimizations, a sufficient number of steepest descent and conjugate gradient iterative cycles were used with the convergence criterion for the average gradient set to 0.01 kcal·mol^−1^·Å^−1^.

### 2.4. Conformational Search

Free inhibitor conformations were derived from their bound conformations in the *E* : *I* complexes by gradual relaxation to the nearest local energy minimum. Then a Monte Carlo search (with an upper limit of 50,000 iterations) for low-energy conformations over all rotatable bonds except those in the rings was carried out using Cerius^2^ molecular modeling package [29]. Two hundred unique conformations were generated for each inhibitor by randomly varying torsion angles of the last accepted conformer by ±15° at 5000 K followed by subsequent energy minimization. During the minimization, a dielectric constant *ε* = 80 was used to account approximately for the dielectric screening effect of hydration upon the generated conformers. The conformer with the lowest total energy was selected and reminimized at *ε* = 4.

### 2.5. Solvation Gibbs Free Energies

The electrostatic component of solvation Gibbs free energy that incorporates also the effects of ionic strength through the solution of nonlinear Poisson-Boltzmann equation [30, 31] was computed by the DelPhi module in Discovery Studio [32]. The program treats the solvent as a continuous medium of high dielectric constant (*ε*_*o*_ = 80) and the solute as a cavity with low dielectric (*ε*_*i*_ = 4) with boundaries linked to the solute's molecular surface, which encloses the solute's atomic charges. The program uses a finite difference method to numerically solve for the molecular electrostatic potential and reaction field around the solute. DelPhi calculations were carried out on a (235 × 235 × 235) cubic lattice grid for the *E* : *I* complexes and free *E* and (65 × 65 × 65) grid for the free *I* with full coulombic boundary conditions. Two subsequent focusing steps led in both cases to a similar final resolution of about 0.3 Å per grid unit at 70% filling of the grid by the solute. Physiological ionic strength of 0.145 mol·dm^−3^, atomic partial charges and radii defined in the CFF91 parameter set [28] and a probe sphere radius of 1.4 Å were used. The electrostatic component of the solvation Gibbs free energy was calculated as the reaction field energy [30, 33–35].

### 2.6. Entropic Term

The vibrational entropy change during the inhibitor binding to the *E* was calculated by normal mode analysis of the inhibitor vibrations using a simplified method of Fischer et al. [36, 37]. In this approach, vibrational analysis of the inhibitor bound at the active site of a “frozen” receptor (*E*) and of the low-energy conformer of the free inhibitor is computed for fully minimized structures using Discover [22] and *T*Δ*S*_vib_ = *TS*_vib_{*I*}_*E*_ − *TS*_vib_{*I*}. It has been shown previously that for small and relatively stiff ligands this method gives a good approximation of the vibrational entropy change of the fully flexible system, that is, including the degrees of freedom of the protein receptor [36, 37]. The *TS*_vib_{*I*} term accounts for vibrational motions of the free inhibitor and represents an indicator of conformational flexibility of the molecule. Namely, low frequency vibrations, which correspond to collective motions of a number of atoms with larger amplitudes, that is, conformational changes, contribute most to this term. Relative values of *T*ΔΔ*S*_vib_ with respect to a reference inhibitor were used to compensate partially for the restricted flexibility of *E*. Although enthalpic contribution to binding affinity is important, the enthalpy/entropy balance is acting more and more as descriptor of selectivity bringing drug optimization to a multidimensional approach [38]. 

### 2.7. Calculation of Binding Affinity

Inhibition constant (*K*_*i*_) of a reversible inhibitor *I* is related to the standard Gibbs free energy (GFE) change of the formation of *E* : *I* complex (Δ*G*_com_) in a solvent. The *K*_*i*_ value can thus be predicted from the complexation GFE Δ*G*_com_ = −RTln*K*_*i*_ assuming the following equilibrium:

(1) {E}aq+{I}aq⟷{E:I}aq,

where  {}_aq_ indicates solvated species. The standard GFE change of reaction (1) can be derived by molecular simulations of the complex and the free reactants:

(2) ΔGcom=G{E:I}−G{E}−G{I}.

In this work, we approximate the exact values of standard GFE for larger systems such as enzyme-inhibitor complexes by the expression [25, 26]:

(3) G{E:I}≈[EMM{E:I}+RT−TStrv{E:I}]+Gsol{E:I}, 

where *E*_MM_{*E* : *I*} stands for the molecular mechanics total energy of the complex (including bonding and nonbonding contributions), and *G*_sol_{*E* : *I*} is the solvation GFE and *TS*_trv_{*E* : *I*} the entropic term:

(4) TStrv{E:I}=TStran⁡{E:I}+TSrot⁡{E:I}+TSvib{E:I}

composed of a sum of contributions arising from translational, rotational, and vibrational motions of *E* : *I*. Assuming that the trans and rot terms for the free *E* and the complex *E* : *I* are approximately equal, we obtain

(5) ΔGcom≈[EMM{E:I}−EMM{E}−EMM{I}]  +[Gsol{E:I}−Gsol{E}−Gsol{I}]+TStran⁡{I}+TSrot⁡{I} −[TSvib{E:I}−TSvib{E}−TSvib{I}]=ΔHMM+TStran⁡{I}+TSrot⁡{I}−ΔTSvib+ΔGsol,

where *TS*_tran⁡_{*I*} and *TS*_*rot*⁡_{*I*} describe the translational and rotational entropy terms of the free inhibitor, and Δ*TS*_vib_ represents the vibrational entropy change upon the complex formation.

Comparison between different inhibitors was done via relative changes in the complexation GFE with respect to a reference inhibitor, *I*_ref_, assuming ideal gas behaviour for the rotational and translational motions of the inhibitors:

(6) ΔΔGcom=ΔGcom(I)−ΔGcom(Iref)=ΔΔHMM−ΔΔTSvib+ΔΔGsol.

The evaluation of relative changes is preferable as it is expected to lead to partial cancellation of errors caused by the approximate nature of the molecular mechanics method as well as solvent and entropic effects description.

### 2.8. Interaction Energy

To calculate MM interaction energy (*E*_int⁡_) between enzyme residues and the inhibitor, a protocol available in Discovery Studio [32] that computes the non-bonded interactions (van der Waals and electrostatic terms) between defined sets of atoms was used. The calculations were performed using the CFF91 force field [28] with a dielectric constant of (5).

### 2.9. Pharmacophore Generation

Bound conformations of inhibitors taken from the models of *E* : *I* complexes were used for building of 3D QSAR pharmacophore by means of Catalyst HypoGen algorithm [39] implemented in Discovery Studio [32]. The top scoring pharmacophore hypothesis was built up in three steps (constructive, subtractive, and optimization steps) from the set of most active inhibitors. Inactive molecules served for definition of the excluded volume. The maximum number of five features allowed by the HypoGen algorithm was selected based on the TMD scaffold and substituents during the pharmacophore generation, namely: hydrophobic aromatic (HYdAr), hydrophobic aliphatic (HYd), hydrogen bond donor, (HBD), hydrogen-bond acceptor (HBA), and ring aromatic (Ar). Adjustable parameters of the protocol were kept at their default values except the uncertainty on the activity, which was set to 1.5 instead of 3. This last choice to bring the uncertainty interval on experimental activity from the large 〈*K*_*i*_/3,3 × *K*_*i*_〉 to a relatively narrow 〈2 × *K*_*i*_/3, 3 × *K*_*i*_/2〉 taking account in this way of the accuracy and homogeneity of the measured inhibitory activities since they are coming from the same work in the same laboratory. During the generation of 10 pharmacophores, the number of missing features was set to 0 and the best one was selected.

## 3. Results and Discussion

A training set of 15 TMDs and validation set of 6 TMVs were selected from 3 series of compounds with measured activities from the same laboratory [14–16]. They are listed in Table 1 and their experimental activity (5–1900 *μ*M) covers a range sufficiently large to build a reliable QSAR model.

### 3.1. QSAR Models

**Figure 2:**
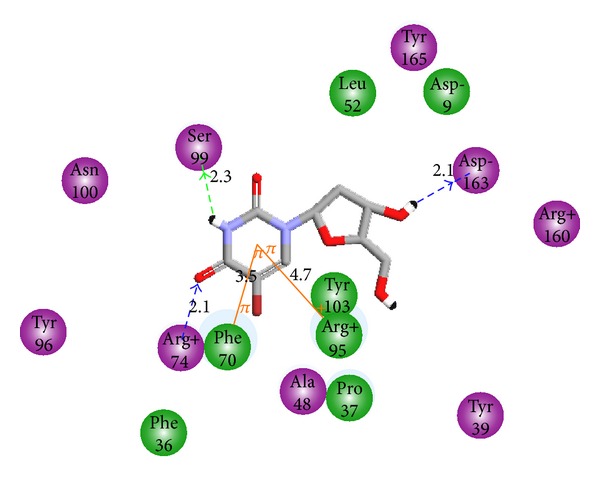
Interaction of TMD4 with active site residues of TMPK*mt. *

**Figure 3:**
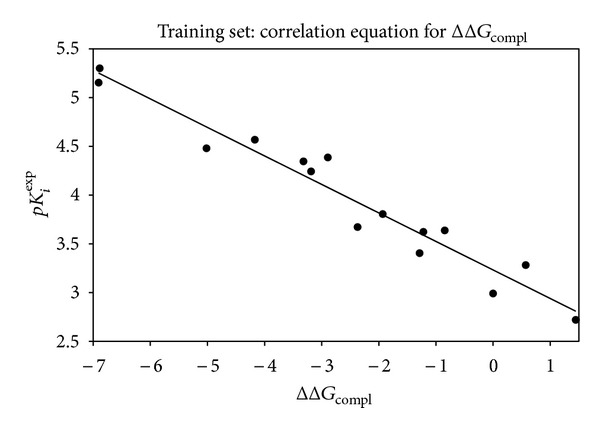
Plot of the correlation equation between *pK*_*i*_^exp⁡^ and relative complexation Gibbs free energy of the training set.

**Figure 4:**
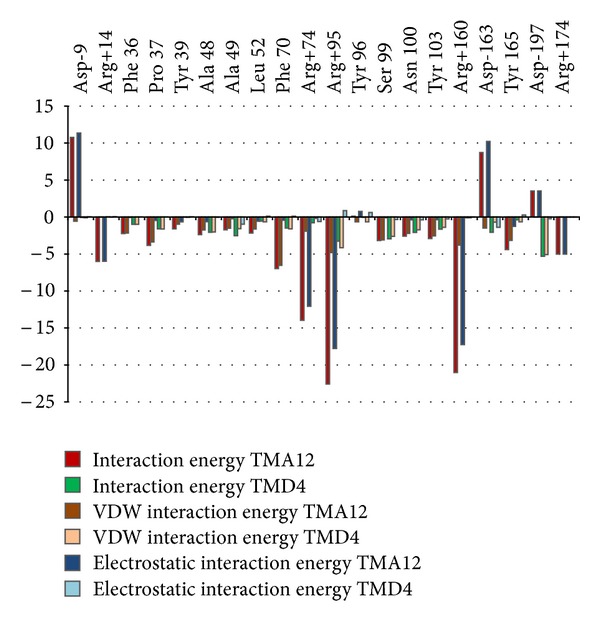
Interaction Energy breakdown comparison for the most active training set compound TMD4 and for the most active designed TMA12.

**Figure 5:**
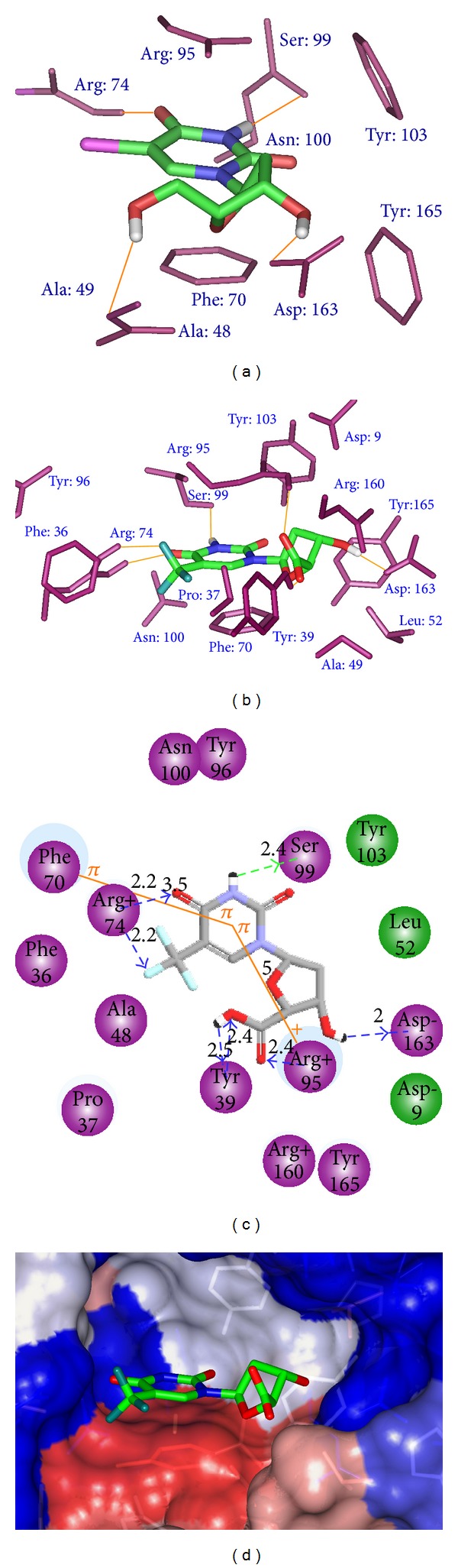
Close-up of TMD4 (a) and TMA12 (b) at the active site of TMPK*mt*. Hydrogen bonds are shown in orange color and the residues are in purple color. Interactions of inhibitor TMA12 at the active site in 2D depiction (c). Connolly surface of the same active site (d). The binding cleft surface is colored according to residue hydrophobicity: red-hydrophobic, blue-hydrophilic and white-intermediate residues.

The relative Gibbs free energy in (2) was computed for the complexes from *in situ* modification of dTMP as described in Material and Methods.  Table 2 lists the Gibbs free energy and its components. The ΔΔ*G*_comp_ reflects the mutual affinity between the enzyme and the inhibitor. Since it is computed from simulations in an approximate way, the consistency of the binding model is evaluated through a regression analysis leading to linear correlation with experimental activity *K*_*i*_. The statistical data of these regressions are illustrated in Figure 3 and listed in Table 3. From the regression equation, the computed ΔΔ*G*_comp_ for a novel compound similar to TMDs is used to predict its enzyme inhibitory activity *K*_*i*_^pred^ provided they share the same binding mode. This process usually can narrow the filter to new lead compounds and save time compared with traditional synthesis approach. From the complexation model structures the computed breakdown of the contribution of each TMPK*mt* active site residue to the interaction with TMD4 (*K*_*i*_ = 5 *μ*M) *E*_int⁡_{TMPK*mt* : TMD4} is compared with the one of TMA12 (*K*_*i*_^pred^ = 0.155 *μ*M) *E*_int⁡_{TMPK*mt* : TMA12} in Figure 4. It comes out clearly from TMD4 to TMA12 that except Tyr39 all the residues involved in strong interaction with TMD4 remain in close contact with TMA12 (Figures 2 and 5(c)) with a noticeable increase of the contributions to *E*_int⁡_ for Phe70, Pro37, Arg74, and Tyr165.

### 3.2. Binding Mode of Inhibitors

**Figure 6:**
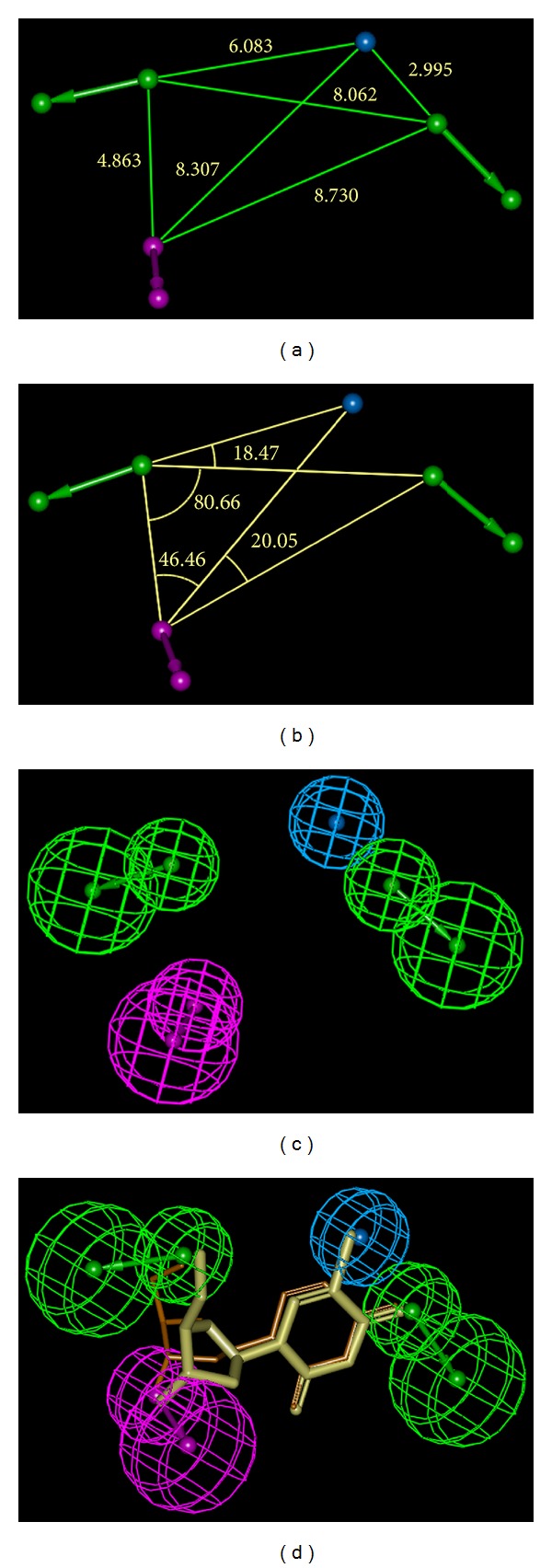
TMPK*mt* inhibition pharmacophore coordinates (a) and (b), features (c) and mapping (d) with TMD4 (purple) and TMD5 (yellow). The correlation plot of predicted versus experimental inhibitory activity is displayed in Figure 7.

The binding modes of TMDs derived from the complexation model are illustrated in Figures 1 and 2. The main interactions at the active site formerly reported from the X-ray structure are conserved except the HBs involving the monophosphate moiety with Arg95, Tyr39, and ribose OH with Asp9 while additional HBs are established with Ala49 and Asp163. The stacking interaction pyrimidine ring, Phe70, is kept along with the cation-*π* interaction with Arg95. This last interaction is the main driving force of the substrate-like orientation of TMDs. The binding mode of dTMP scaffold as reported from X-ray structures is reproduced by our model based on thymidine scaffold making enrichment of interactions by new substitutions straightforward provided the accuracy of our predictive equation is sufficiently stable to cover the nanomolar range with the assumption that structurally similar ligands bind in a similar fashion. A quick analysis of the training set shows that halogen in the 5-position on pyrimidine ring is not the main feature for potency confirmed by the relatively low activity of TMD2-3. At the other ends in 5′-position only N3 bring substantial increase of potency. These two positions are of great importance for activity as indicated by the pharmacophore features in Figure 6. It is easy to appreciate the weight of each feature since CH3 in 5-position in a majority of inhibitors did not improve the inhibitory potency resulting in a 5-fold decrease in TMD15 (27 *μ*M) compared with Br in TMD4 (5 *μ*M). The cation-*π* interaction between the pyrimidine ring and Agr95 (32% of *E*_int⁡_{TMPK*mt* : TMA12}) is conserved along with the *π*-*π* stacking involving Phe70 (10% of *E*_int⁡_{TMPK*mt* : TMA12}) as we can see in Figure 5(c).

### 3.3. Pharmacophore Model of Inhibitory Activity

The 3D QSAR PH4 generation process follows three main steps, the constructive, the subtractive, and the optimization steps. The constructive phase of HypoGen automatically selected as lead compounds (5 ≤ *K*_*i*_ ≤ 7 *μ*M) for which (5 × 1.5 − *K*_*i*_/1.5 > 0) TMD4 and TMD5 using these top two to generate all the starting PH4 features and retaining only those fitting the remaining leads. The subtractive phase in which inactive compounds (log(*K*_*i*_)–log⁡(5) > 3.5) are used to remove features that map more than 50% of them retained representatives of all the selected five features. In the optimization phase using the simulated annealing algorithm the highest scoring PH4s are retained according to their probability function-based cost. A total of 10 hypotheses were generated all displaying four features. The costs range from 55.1 (Hypo1) to 75.2 (Hypo10). This short gap supports the homogeneity of the hypotheses and the adequacy of the training set. The fixed cost (45.9) is lower than the null cost (157.4) by Δ = 111.5. This difference is a major quality indicator of the PH4 predictability; Δ > 70 corresponds to an excellent chance or a probability higher than 90% that the model represents a true correlation [32]. To be statistically significant the hypothesis has to be as closer as possible to the fixed and as further as possible from the null cost, for this set of 10 hypotheses Δ ≥  82.2 attests the quality of the model. The standard indicators like the root-mean-square deviations (RMSD) and the correlation coefficients (*R* ^2^) range from 1.126 to 2.036 and from 0.96 to 0.88, respectively. Due to the closed values for the whole set of PH4s, the first hypothesis (Hypo1) has been retained for further analysis. 

The data for the set of hypotheses (costs, RMSD, *R*) are listed in Table 5. The statistical data of Hypo1; *pK*_*i*_  =  1.0206  ×  *K*_*i*_^pred^ − 0.0832  (*n* = 15, *R*² = 0.92, *R*_XV_^2^ = 0.91, *F* = 132.1, *σ* = 0.231 at a significance level >95%) are illustrated by the correlation plot in Figure 6 showing also the geometry of the pharmacophore and TMD4 and TMD5 mapping to it. To check the consistency of the model, we predicted the activity (*pK*_*i*_) of the validation set (TMVs: TMV1-1.65; TMV2-1.16, TMV3-1.56, TMV4-1.26, TMV5-0.82, TMV6-1.02) all close to one except TMV1 and TMV3. 

The randomization validation (Fisher method) of the PH4 model by CatScramble algorithm in Catalyst has been carried out from 49 random runs according to the 98% confidence level selected creating each time 10 valid hypotheses. Finally none of them was as predictive as the 10 lowest listed in Table 5, their costs ranged from 56 to 87 for the closest and from 116 to 135 for the highest; neither their correlation nor RMSD were better. To recapitulate there is 98% probability that the selected hypotheses provide a model at the same level of predictability as the complexation one for the biological activity of TMDs. The design of the new analogues of this work is based partially on the hydrophobic feature representing CH_3_ group of the thymine ring. It was also the unique feature observed by Gopalakrishnan et al. [12] in a virtual screening approach for TMPK*mt* inhibitors. The difference between the null and the fixed cost in their case was 45 lower than the 70 threshold needed for a good predictability quality. Three HBA and one HBD features were added to the Hyd one in the model. The interfeature distances are different; in our model, the closest HBA to the Hyd is at 2.995 Å instead of 4.43 Å, 6.083 instead of 6.88 Å. It has to be indicated also that the HBD feature is related to the hydroxyl group of the ribose ring and accordingly the bound conformation being different from the free ligand one differences is expected from both pharmacophores and the nonavailability of the angle from their PH4 model makes a full comparison difficult.

The new inhibitors have been designed in respect to the PH4. A recent 3D pharmacophore study of substituted *α*-Thymidine Analogues inhibitors of TMPK*mt* using optimized receptor-independent (RI) 4D QSAR formalism [40] identified five Grid Cell Occupancy Descriptors (GCODs) for close interaction with Arg95 and Tyr103-Arg74-Asp163, Asp9 and Arg95-Ala35, Phe36, Pro37, and Arg160-Met66 and Phe70. One is inhibition-enhancing (close to the pyrimidine ring carbonyl in 4-position) and the other four inhibition diminishing. From this list only Ala35 and Met66 are missing in our QSAR model list of active site residues in close interaction with the inhibitors. The positions of the GCODs correspond globally with our generated PH4 model features.

### 3.4. New Inhibitors

The novel analogues are based mostly on the substitution in the 5-position on the pyrimidine ring, 2′ and 3′ of the ribose. More global replacement such as ribose → cyclopentane and a capping carboxylate at the 5′ end. They are listed in Table 4 (see also Figure 5), the most active ones reaching picomolar concentration range. 

#### 3.4.1. Substitution at the 5′ End (R_4_)

Keeping all the remaining moiety unchanged, the 5′ hydroxyl group in the training set has been replaced with various (TMA1-6, TMA18) polar groups among which the carboxylate COO^−^ performed the best but with a fivefold decrease of potency (*K*_*i*_ = 24 nM) from TMD4. As we can see from Figure 5(b), the carboxylate interacts through two HB with Arg95 and Tyr39 bringing substantial affinity towards TMPK*mt*. The former OH was not involved in any HB (Figure 2). About this additional HB with Tyr39 it is interesting to point out, among the few differences of the binding sites of TMPK*mt* and TMPK*h* (the human corresponding enzyme-PDB code 1E99) where Arg14, Tyr39 and Asn100 are replaced in the last one by Ser20, Arg45 and Gly102, respectively [41], the crucial opportunity to exploit this structural and functional difference (interaction with Tyr39) to design selective inhibitors for TMPK*mt*. Recent attempts of extension by replacement of the carboxylate COO^−^ with other groups resulting in 5′-modified thymidines are promising [42].

#### 3.4.2. Substitution at the 5-Position of the Pyrimidine Ring (R_1_)

Keeping the carboxylate group and the ribose ring (R_2_=H and R_3_=OH) in place, replacement of bromine at the 5-position (TMA7-17) resulted in noticeable increase of potency for TMA12 R_1_=CF_3_-*K*_*i*_ = 0.155 nM, TMA16 R_1_=CH_2_–NH–CHO-*K*_*i*_ = 0.155 nM, and TMA17 R_1_=NH–COCH_3_-*K*_*i*_ = 0.341. The trifluoromethyl group is in a supplemental HB contact with Arg74 adding to the cation-*π* (Arg95) and *π*-*π* interaction (Phe70) of the pyrimidine ring (Figure 5(b)). 

#### 3.4.3. Substitution at the 3′ Position of the Ribose Ring (R_3_)

In order to improve the predicted potency for TMA12, 16 and 17 replacement of OH by NH_2_ on the ribose ring does not result in any increase in predicted inhibitory activity, the best one TMA37 *K*_*i*_ = 1 nM being sixfold less potent than TMA12. Any other design modification, for example replacement of ribose by a cyclopentane, led to a decrease of potency (TMA19–27 and TMA41–47).

Figures 5(b) and 5(c) show the main interactions supporting the affinity of TMA12 in comparison with those coming out from our QSAR model for TMDs. The Connolly surface presented in Figure 5(d) suggests further design opportunities such as bicyclic 2′,3′-ring inhibitors of TMPK*mt* [20] and extension of the carboxylate end to derivatives carrying a naphtholactam or naphthosultam moiety at position 4 of a (Z)-butenyl chain [43]. The design of this class of inhibitors requires appropriate training set available (*K*_*i*_: 0.42–0.75 *μ*M). We are building the QSAR model and new analogues will be released in due course [44]. 

The contribution of the residues specific to TMPK*mt* to *E*_int⁡_{TMPK*mt* : TMA12} (Figure 4) globally 15% breakdown into Arg14 (9%), Tyr39 (2%) and Asn100 (4%) is encoura-ging compared with their value for *E*_int⁡_{TMPK*mt* : TMD4} globally 3.5% limited to Asn100 (3%) for the most active training set inhibitor.

## 4. Conclusions

The interaction model built based on complexation methodology and validated by a PH4 analysis provided structural information helpful in the design of new analogues. They are structurally close to reported dTMP analogues. Here a carboxylate in place of 5′-position hydroxyl increased their inhibitory potency reaching low to subnanomolar range (1–0.155 nM). One of the most potent new analogue TMA12 is HB bonded to the selective Tyr39 making the best of TMAs a promising set for synthesis and evaluation. A more systematic search through screening of combinatorial library and subsequent evaluation on enzymatic assay will lead to more potent analogues.

## Figures and Tables

**Figure 7:**
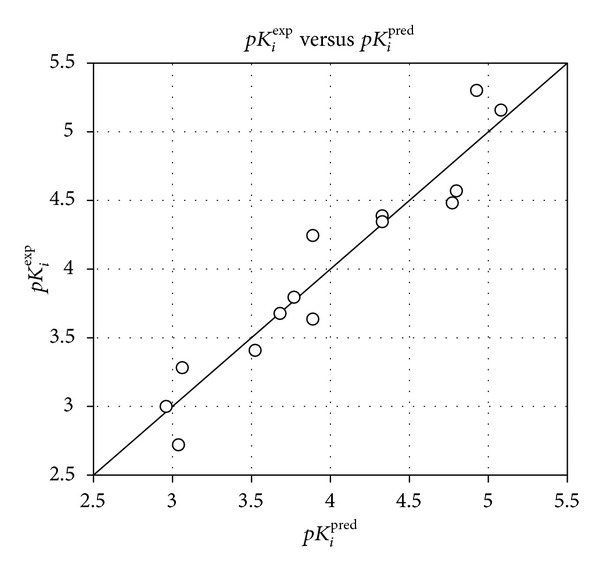
Plot of the correlation equation between pharmacophore (PH4) predicted and experimental inhibitory activity of the training set TMDs.

**Table 1:** Training and validation sets of TMD inhibitors for QSAR model.

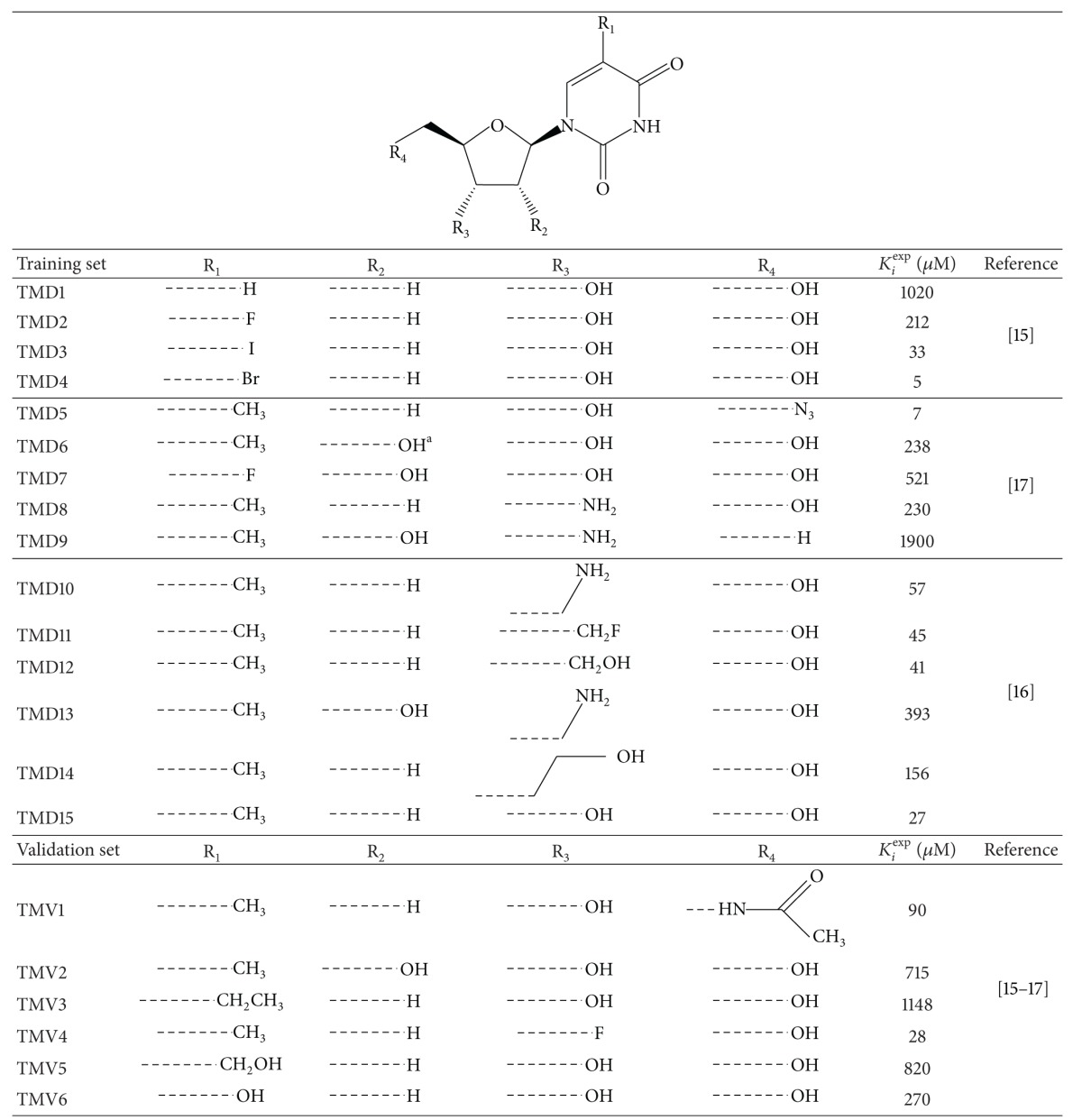

^a^Hydroxyl in *β* position.

**Table 2:** Complexation energy and its components for the training set of TMPK*mt* inhibitors: TMD1–TMD15.

Training set^a^	*M*_*w*_^b^	ΔΔ*H*_MM_^c^	ΔΔ*G*_sol_^d^	ΔΔTS_vib_^e^	ΔΔ*G*_comp_^f^	K_i_^exp⁡^g^^
	(g · mol^−1^)	(kcal · mol^−1^)	(kcal · mol^−1^)	(kcal · mol^−1^)	(kcal · mol^−1^)	(*μ*M)
TMD1	228	0	0	0	0	1020
TMD2	246	−3.942	3.537	−1.968	−2.373	212
TMD3	354	−4.695	1.873	−2.196	−5.017	33
TMD4	306	−1.631	−3.514	−1.742	−6.887	5
TMD5	333	−5.880	1.212	−2.238	−6.906	7
TMD6	258	−3.461	3.613	−1.371	−1.220	238
TMD7	262	−5.157	7.239	−1.510	0.571	521
TMD8	242	3.496	−4.720	0.378	−0.847	230
TMD9	242	1.916	−1.255	0.786	1.447	1900
TMD10	256	−0.110	−3.672	−0.390	−4.172	57
TMD11	258	−21.643	22.028	−3.572	−3.187	45
TMD12	256	−1.323	0.216	−2.213	−3.320	41
TMD13	272	3.727	−3.906	−2.715	−2.894	393
TMD14	270	−3.988	6.637	−3.934	−1.286	156
TMD15	242	−6.706	9.914	−5.140	−1.932	27

Validation set	*M*_*w*_	ΔΔ*H*_MM_	ΔΔ*G*_sol_	ΔΔTS_vib_	ΔΔG_comp _	*pK*_*i*_^pred^/*pK*_*i*_^exp⁡^
	[g · mol^−1^]	[kcal · mol^−1^]	[kcal · mol^−1^]	[kcal · mol^−1^]	[kcal · mol^−1^]	

TMV1	283	−1.641	−0.724	−1.807	−4.172	1.100
TMV2	258	−4.852	6.608	−0.372	1.385	0.899
TMV3	256	−4.166	2.479	−0.282	−1.969	1.296
TMV4	244	2.350	−4.156	−1.145	−2.951	0.899
TMV5	258	−4.310	3.609	−0.527	−1.228	1.164
TMV6	244	−3.999	2.689	−1.471	−2.780	1.134

^a^For the chemical structures of the training set of inhibitors see Table 1.

^b^ *M*_*w*_ is the molecular mass of the inhibitor.

^c^ΔΔ*H*_MM_ is the relative enthalpic contribution to the Gibbs free energy change related to the protease-inhibitor complex formation derived by molecular mechanics (MM): ΔΔ*H*_MM_≅[*E*_MM_{PR:TMDx} − *E*_MM_{TMDx}]−[*E*_MM_{PR:TMD1} − *E*_MM_{TMD1}], TMD1—is the reference inhibitor;

^d^ΔΔ*G*_solv_ is the relative solvation Gibbs free energy contribution to the Gibbs free energ**y **change related to protease-inhibitor complex formation: ΔΔ*G*_solv_ = [*G*_solv_{PR:TMDx} − *G*_solv_{TMDx}] − [*G*_sol_{PR:TMD1} − *G*_sol_{TMD1}];

^e^−ΔΔTS_vib_ is the relative entropic contribution of the inhibitor to the Gibbs free energy related to protease-inhibitor complex formation: ΔΔTS_vib_ = [TS_vib_{TMDx}_PR_ − TS_vib_{TMDx}]−[TS_vib_{TMD1}_PR_ − TS_vib_{TMD1}];

^f^ΔΔG_comp _ is the relative Gibbs free energy change related to the enzyme-inhibitor complex formation: ΔΔG_comp⁡_≅ΔΔ*H*_MM_ + ΔΔ*G*_solv_ − ΔΔTS_vib_.

^g^ *K*_*i*_^exp⁡^ is the experimental TMPK*mt* inhibition constant obtained from [15–17].

^h^Ratio of predicted and experimental inhibition constants *pK*_*i*_^pre^/*pK*_*i*_^exp⁡^ · *K*_*i*_^pre^ was predicted from computed ΔΔG_comp_ using the regression equation for TMPK*mt* shown in Table 3.

**Table 3:** Statistical information on regression analysis of correlation for the training set between ΔΔ*G*_comp_ and experimental activities (*pK*_*i*_) respectively against TMPK*mt*.

*pK*_*i*_ = −0.2924 ΔΔ*G*_comp_ + 3.234	

Statistical data of regression analysis	ΔΔ*G*_comp_

Number of compounds *n*	15
Squared correlation coefficient of regression *R* ^2^	0.944
LOO cross-validated squared correlation coefficient *R*_XV_^2^	0.939
Standard error of the regression σ	0.184
Statistical significance of regression, Fisher *F*-test *F*	218.3
Level of statistical significance α	>95%
Range of activity of *K*_*i*_ (*μ*M)	5–1900

**Table 4:** Designed analogs with predicted activity.

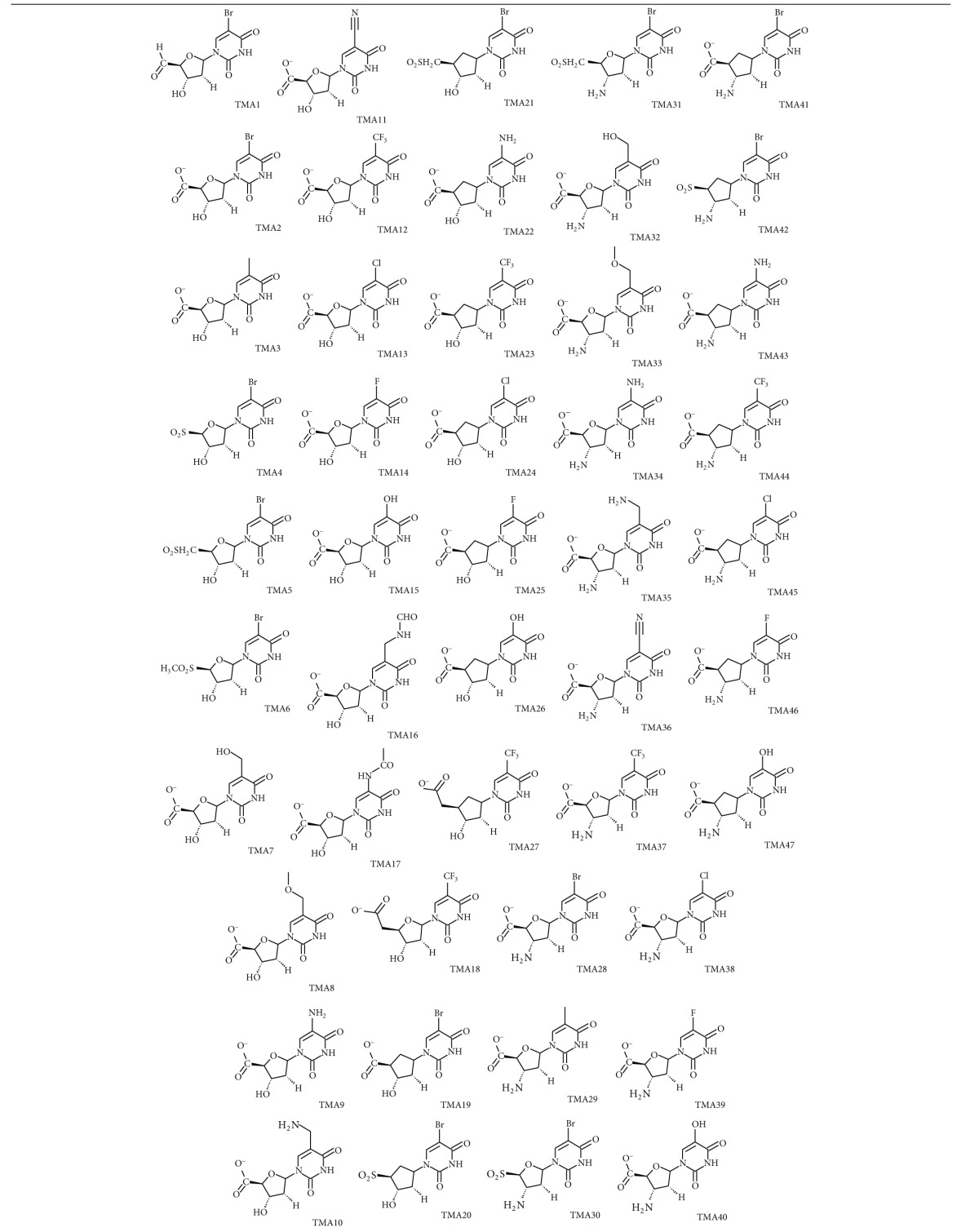 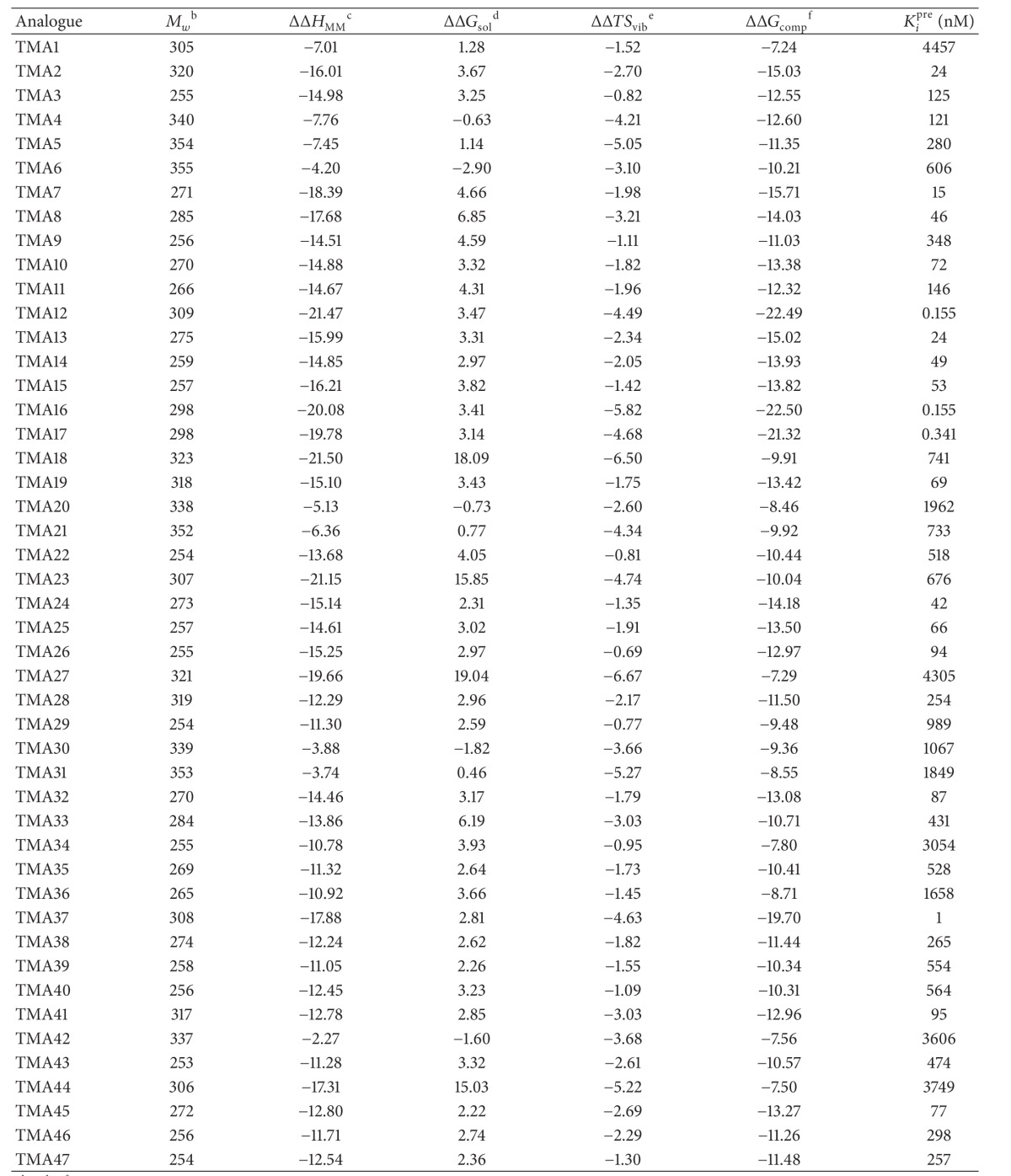

^a, b, c, d, e, f^See Table 2.

**Table 5:** Output parameters of the 10 generated PH4 Hypotheses after CatScramble validation procedure for TMPK*mt* inhibitors listing RMSD, total cost and *R* correlation coefficient.

Hypothesis	RMSD	*R *	Total costs
Hypo1	1.126	0.964	55.1
Hypo2	1.207	0.958	57.1
Hypo3	1.477	0.936	61.1
Hypo4	1.802	0.904	68.7
Hypo5	1.831	0.901	69.7
Hypo6	1.941	0.888	72.3
Hypo7	1.975	0.883	73.7
Hypo8	2.016	0.878	74.4
Hypo9	2.023	0.877	75.2
Hypo10	2.036	0.876	75.2
Fixed cost	0	1.0	45.9
Null cost	4.213	0	157.4

## References

[1] Nunn P, Falzon D WHO information note on tuberculosis and pandemic influenza A, (H1N1). http://www.who.int/entity/tb/features_archive/h1n1_and_tuberculosis.pdf.

[2] Korenromp EL, Bierrenbach AL, Williams BG, Dye C (2009). The measurement and estimation of tuberculosis mortality. *International Journal of Tuberculosis and Lung Disease*.

[3] Murphy DJ, Brown JR (2008). Novel drug target strategies against *Mycobacterium tuberculosis*. *Current Opinion in Microbiology*.

[4] global tuberculosis control—surveillance, planning, financing. http://www.who.int/tb/publications/global_report/2008/en/index.html.

[5] Raviglione MD, Snider DE, Kochi A (1995). Globa epidemiology of tuberculosis. Morbidity and mortality of a worldwide epidemic. *The Journal of the American Medical Association*.

[6] WHO Global Tuberculosis Programme Tuberculosis. http://www.who.int/mediacentre/factsheets/fs104/en/.

[7] Dye C, Maher D, Weil D, Espinal M, Raviglione M (2006). Targets for global tuberculosis control. *International Journal of Tuberculosis and Lung Disease*.

[8] Lång H, Quaglio G, Olesen OF (2010). Tuberculosis research in the European Union: past achievements and future challenges. *Tuberculosis*.

[9] Boshoff HIM, Xu X, Tahlan K (2008). Biosynthesis and recycling of nicotinamide cofactors in *Mycobacterium tuberculosis*: an essential role for NAD in nonreplicating bacilli. *Journal of Biological Chemistry*.

[10] Lavie A, Ostermann N, Brundiers R (1998). Structural basis for efficient phosphorylation of 3′-azidothymidine monophosphate by *Escherichia coli* thymidylate kinase. *Proceedings of the National Academy of Sciences of the United States of America*.

[11] Haouz A, Vanheusden V, Munier-Lehmann H (2003). Enzymatic and structural analysis of inhibitors designed against *Mycobacterium tuberculosis* thymidylate kinase: new insights into the phosphoryl transfer mechanism. *Journal of Biological Chemistry*.

[12] Gopalakrishnan B, Aparna V, Jeevan J, Ravi M, Desiraju GR (2005). A virtual screening approach for thymidine monophosphate kinase inhibitors as antitubercular agents based on docking and pharmacophore models. *Journal of Chemical Information and Modeling*.

[13] Ursby T, Weik M, Fioravanti E, Delarue M, Goeldner M, Bourgeois D (2002). Cryophotolysis of caged compounds: a technique for trapping intermediate states in protein crystals. *Acta Crystallographica Section D*.

[14] Li De La Sierra I, Munier-Lehmann H, Gilles AM, Bârzu O, Delarue M (2001). X-ray structure of TMP kinase from *Mycobacterium tuberculosis* complexed with TMP at 1.95 Å resolution. *Journal of Molecular Biology*.

[15] Pochet S, Dugué L, Labesse G, Delepierre M, Munier-Lehmann H (2003). Comparative study of purine and pyrimidine nucleoside analogues acting on the thymidylate kinases of *Mycobacterium tuberculosis* and of humans. *ChemBioChem*.

[16] Vanheusden V, Van Rompaey P, Munier-Lehmann H, Pochet S, Herdewijn P, Van Calenbergh S (2003). Thymidine and thymidine-5′-O-monophosphate analogues as inhibitors of *Mycobacterium tuberculosis* thymidylate kinase. *Bioorganic and Medicinal Chemistry Letters*.

[17] Gasse C, Douguet D, Huteau V, Marchal G, Munier-Lehmann H, Pochet S (2008). Substituted benzyl-pyrimidines targeting thymidine monophosphate kinase of *Mycobacterium tuberculosis*: synthesis and in vitro anti-mycobacterial activity. *Bioorganic and Medicinal Chemistry*.

[18] Van Daele I, Munier-Lehmann H, Hendrickx PMS (2006). Synthesis and biological evaluation of bicyclic nucleosides as inhibitors of *M. tuberculosis* thymidylate kinase. *ChemMedChem*.

[19] Familiar O, Munier-Lehmann H, Negri A (2008). Exploring acyclic nucleoside analogues as inhibitors of *Mycobacterium tuberculosis* thymidylate kinase. *ChemMedChem*.

[20] Frecer V, Seneci P, Miertus S (2011). Computer-assisted combinatorial design of bicyclic thymidine analogs as inhibitors of *Mycobacterium tuberculosis* thymidine monophosphate kinase. *Journal of Computer-Aided Molecular Design*.

[21] Berman HM, Westbrook J, Feng Z (2000). The protein data bank. *Nucleic Acids Research*.

[22] Insight-II and discover molecular modeling and simulation package.

[23] Frecer V, Kabeláč M, De Nardi P, Pricl S, Miertuš S (2004). Structure-based design of inhibitors of NS3 serine protease of hepatitis C virus. *Journal of Molecular Graphics and Modelling*.

[24] Frecer V, Jedinak A, Tossi A (2005). Structure based design of inhibitors of aspartic protease of HIV-1. *Letters in Drug Design and Discovery*.

[25] Frecer V, Berti F, Benedetti F, Miertus S (2008). Design of peptidomimetic inhibitors of aspartic protease of HIV-1 containing -PheΨPro- core and displaying favourable ADME-related properties. *Journal of Molecular Graphics and Modelling*.

[26] Dali B, Keita M, Megnassan E, Frecer V, Miertus S (2012). Insight into selectivity of peptidomimetic Inhibitors with modified statine core for plasmepsin II of *Plasmodium falciparum* over human cathepsin D. *Chemical Biology and Drug Design*.

[27] Megnassan E, Keita M, Bieri C, Esmel A, Frecer V, Miertus S (2012). Design of novel dihydroxynaphthoic acid inhibitors of *Plasmodium falciparum* lactate dehydrogenase. *Medicinal Chemistry*.

[28] Maple JR, Hwang MJ, Stockfish TP (1994). Derivation of class II force fields. 1. Methodology and quantum force field for the alkyl functional group and alkane molecules. *Journal of Computational Chemistry*.

[29] Cerius2 life sciences molecular simulation software.

[30] Gilson MK, Honig B (1991). The inclusion of electrostatic hydration energies in molecular mechanics calculations. *Journal of Computer-Aided Molecular Design*.

[31] Rocchia W, Sridharan S, Nicholls A, Alexov E, Chiabrera A, Honig B (2002). Rapid grid-based construction of the molecular surface and the use of induced surface charge to calculate reaction field energies: applications to the molecular systems and geometric objects. *Journal of Computational Chemistry*.

[32] Discovery studio molecular modeling and simulation program.

[33] Böttcher CJF (1973). *Theory of Electric Polarization*.

[34] Miertus S, Scrocco E, Tomasi J (1981). Electrostatic interaction of a solute with a continuum. A direct utilizaion of AB initio molecular potentials for the prevision of solvent effects. *Chemical Physics*.

[35] Frecer V, Miertus S (1992). Polarizable continuum model of solvation for biopolymers. *International Journal of Quantum Chemistry*.

[36] Fischer S, Smith JC, Verma CS (2001). Dissecting the vibrational entropy change on protein/ligand binding: Burial of a water molecule in bovine pancreatic trypsin inhibitor. *Journal of Physical Chemistry B*.

[37] Schwarzl SM, Tschopp TB, Smith JC, Fischer S (2002). Can the calculation of ligand binding free energies be improved with continuum solvent electrostatics and an ideal-gas entropy correction?. *Journal of Computational Chemistry*.

[38] Freire E (2008). Do enthalpy and entropy distinguish first in class from best in class?. *Drug Discovery Today*.

[39] Li H, Sutter J, Hoffmann R, Güner OF (2000). HypoGen: an automated system for generating 3D predictive pharmacophore models. *Pharmacophore Perception, Development and Use in Drug Design*.

[40] Andrade CH, Pasqualoto KFM, Ferreira EI, Hopfmger AJ (2009). Rational design and 3D-pharmacophore mapping of 5′-thiourea- substituted *α*-thymidine analogues as mycobacterial TMPK inhibitors. *Journal of Chemical Information and Modeling*.

[41] Ostermann N, Lavie A, Padiyar S (2000). Potentiating AZT activation: structures of wild-type and mutant human thymidylate kinase suggest reasons for the mutants’ improved kinetics with the HIV prodrug metabolite AZTMP. *Journal of Molecular Biology*.

[42] Toti KS, Verbeke F, Risseeuw MDP, Frecer V, Munier-Lehmann H, Van Calenbergh S (2013). Synthesis and evaluation of 5′-modified thymidines and 5-hydroxymethyl-2′-deoxyuridines as *Mycobacterium tuberculosis* thymidylate kinase inhibitors. *Bioorganic and Medicinal Chemistry*.

[43] Familiar O, Munier-Lehmann H, Negri A (2008). Exploring acyclic nucleoside analogues as inhibitors of *Mycobacterium tuberculosis* thymidylate kinase. *ChemMedChem*.

[44] Kumar A, Megnassan E, Siddiqi I, Frecer V, Miertus S Rationa design of inhibitors of *Mycobacterium tuberculosis* Thymidine monophosphate kinase.

